# All American Dental Degrees Rejected in Great Britain

**Published:** 1893-07

**Authors:** 


### ALL AMERICAN DENTAL DEGREES REJECTED IN GREAT BRITAIN.

The Medical Council of Great Britain has decided that the dental diplomas from Harvard and Michigan Universities shall, hereafter, be refused registration. We will print the full text of the official letter in our next issue.

